# Evidence-based clinical practice guidelines for functional dyspepsia 2021

**DOI:** 10.1007/s00535-021-01843-7

**Published:** 2022-01-21

**Authors:** Hiroto Miwa, Akihito Nagahara, Akihiro Asakawa, Makoto Arai, Tadayuki Oshima, Kunio Kasugai, Kazuhiro Kamada, Hidekazu Suzuki, Fumio Tanaka, Kazunari Tominaga, Seiji Futagami, Mariko Hojo, Hiroshi Mihara, Kazuhide Higuchi, Motoyasu Kusano, Tomiyasu Arisawa, Mototsugu Kato, Takashi Joh, Satoshi Mochida, Nobuyuki Enomoto, Tooru Shimosegawa, Kazuhiko Koike

**Affiliations:** 1 Guidelines Committee for Creating and Evaluating the “Evidence-Based Clinical Practice Guidelines for Functional Dyspepsia”, The Japanese Society of Gastroenterology, 6F Shimbashi i-MARK Building, 2-6-2 Shimbashi, Minato-ku, Tokyo, 105-0004 Japan; 2 Division of Gastroenterology and Hepatology, Department of Internal Medicine, Hyogo College of Medicine, 1-1 Mukogawa-cho, Nishinomiya, Hyogo 663-8501 Japan

**Keywords:** Dyspepsia, Guideline, Proton pump inhibitor, Prokinetics, Antianxiety drug, Antidepressant, Japanese traditional medicine, *H*. *pylori* eradication treatment, *H*. *pylori*-associated dyspepsia, Algorithm, Chronic gastritis

## Abstract

**Background:**

Functional dyspepsia (FD) is a disorder that presents with chronic dyspepsia, which is not only very common but also highly affects quality of life of the patients. In Japan, FD became a disease name for national insurance in 2013, and has been gradually recognized, though still not satisfactory. Following the revision policy of Japanese Society of Gastroenterology (JSGE), the first version of FD guideline was revised this time.

**Method:**

Like previously, the guideline was created by the GRADE (grading of recommendations assessment, development and evaluation) system, but this time, the questions were classified to background questions (BQs, 24 already clarified issues), future research questions (FRQs, 9 issues cannot be addressed with insufficient evidence), and 7 clinical questions that are mainly associated with treatment.

**Results and Conclusion:**

These revised guidelines have two major features. The first is the new position of endoscopy in the flow of FD diagnosis. While endoscopy was required to all cases for diagnosis of FD, the revised guidelines specify the necessity of endoscopy only in cases where organic disease is suspected. The second feature is that the drug treatment options have been changed to reflect the latest evidence. The first-line treatment includes gastric acid-secretion inhibitors, acetylcholinesterase (AChE) inhibitors (acotiamide, a prokinetic agent), and Japanese herbal medicine (rikkunshito). The second-line treatment includes anxiolytics /antidepressant, prokinetics other than acotiamide (dopamine receptor antagonists, 5-HT4 receptor agonists), and Japanese herbal medicines other than rikkunshito. The patients not responding to these treatment regimens are regarded as refractory FD.

## Introduction

Many people suffer from dyspeptic symptoms, but the cause is often unclear. Functional dyspepsia (FD) is a disorder that presents with chronic manifestation of such symptoms. Although FD is common, the disease name “functional dyspepsia” had not been widely used in routine medical practice because the concept of FD is relatively new and the name is difficult to understand. However, awareness of FD has been increasing gradually. Factors contributing to the increasing awareness include heightened concerns about quality of life (QOL) that have accompanied improved standards of living in Japan, concern that the stress associated with the growing complexity of modern life is contributing to the occurrence of dyspepsia, and the recognition of “functional dyspepsia” as a disease name for national insurance billing purposes in May 2013. In this context, clinical practice guidelines for FD were published by the Japanese Society of Gastroenterology (JSGE) in 2014, and the number of copies of those guidelines sold far exceeded that of any other guidelines published by the JSGE, indicating a high level of interest in FD.

In view of the rapid progress in medical research and clinical practice, JSGE has adopted a so-called sunset rule, which is a rule that clinical practice guidelines be revised every 5 years. In April 2017, on the basis of that rule, the Board of Directors of JSGE made the decision to revise the clinical practice guidelines for FD, and work on the revised guidelines was begun by the Guidelines Creation Committee. Like the previous version of the guidelines, the revised guidelines were also created using the GRADE (grading of recommendations assessment, development and evaluation) system, but this time, it was decided to make the guidelines easier to understand by limiting the number of clinical questions (CQs). Therefore, issues that had already been clarified were handled as background questions (BQs) and questions for which a clear answer was not possible because of insufficient evidence were treated as future research questions (FRQs). The resulting guidelines were created from 24 BQs, 9 FRQs, and 7 CQs. The literature was searched systematically by the Japan Medical Library Association, with the search period being from 1983 to July 2020. The committee members discussed and finalized the proposed BQs, CQs, and FRQs and then voted to determine the recommendation grades. Next, the manuscript was checked and revised by the Evaluation Committee, and the revised manuscript was subjected to public comment by the members of JSGE. After final revision on the basis of the members’ comments, the Japanese manuscript was completed in January 2021 and published in April 2021.

The revised guidelines have two major features. The first is the new position of endoscopy in the flow of FD diagnosis. Whereas previously organic disease had to be excluded by endoscopy to diagnose FD (the disease name “functional dyspepsia” could not be used for national insurance billing unless endoscopy had been performed), the revised guidelines specify that endoscopy should be performed in all cases where organic disease is suspected. Clinical determination of whether organic disease is suspected and endoscopy is necessary has been left to the judgment and discretion of the physician. Formerly, endoscopy had been required for a diagnosis of FD even in patients who were negative for *Helicobacter pylori*, patients as young as 20 years of age, and patients who had been screened for stomach cancer in the previous 6 months. The revised guidelines, however, have been changed to specify that rather than endoscopy being performed indiscriminately, the need for it should be determined for each patient depending on the patient’s physical findings, history (family, disease, tests), and other relevant factors. By eliminating unnecessary tests, this change is expected to bring many benefits, including reducing the physical and financial burden on patients, allowing their treatment to begin sooner, and helping control the cost of medical care to society.

The second major feature of the revised guidelines is the drug treatment options have been changed to reflect the latest evidence. Gastric acid-secretion inhibitors and prokinetic agents have been divided into different classes and a recommendation grade has been assigned to each class. The classes of gastric acid-secretion inhibitors are proton pump inhibitors (PPIs), H2-receptor antagonists (H2RAs), and potassium-competitive acid blockers (P-CABs), and the classes of prokinetic agents are acetylcholinesterase (AChE) inhibitors, dopamine receptor antagonists, and serotonin-4 (5-HT4) receptor agonists. Another important difference from the previous version of the guidelines is that the Japanese herbal medicine rikkunshito, for which there is abundant evidence, has been assigned a recommendation grade higher than that of other herbal medicines. These changes are in line with the spirit of JSGE guidelines, which is to build treatment systems based on evidence. As an aide for implementing the revised guidelines, an algorithm for the diagnosis and treatment of FD that reflects the new position of endoscopy and the recommendation grades of the available treatments has been prepared.

This article summarizes the Japanese guidelines, with particular focus on the treatment section. To prepare the guidelines, specialists in relevant fields in Japan collected evidence, discussed it, and then voted on it, so the guidelines are based on the current situation in Japan. Among the diverse countries and regions of the world, there are great differences in disease occurrence, the medical resources available, and the medical environments, as well as in lifestyles and cultures. Therefore, the authors think that standardization of medical care for dyspepsia in each country or region should be done in a manner appropriate for the local conditions. Nevertheless, we hope that our guidelines will be able to serve as a useful reference in the standardization of the diagnosis and treatment of FD in a wide variety of countries and regions.

## Algorithm

**Fig. 1:**
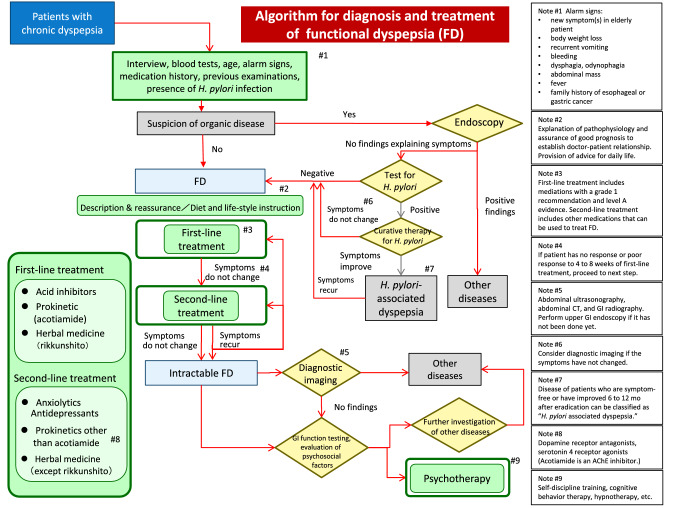
Algorithm for the diagnosis and treatment of functional dyspepsia (FD)

Figure 1 shows the algorithm (flowchart) for the diagnosis and treatment of FD. The algorithm represents the consensus opinion of the members of the Guidelines Creating Committee and emphasizes strength of recommendation and level of evidence.

In creating the algorithm, the committee kept in mind two important features of these revised guidelines. The first is the new recommendation that endoscopy should be performed in any patient suspected of having organic disease. To emphasize that the physician may diagnose FD directly in cases where organic disease is not suspected from the medical history, *H*. *pylori* infection status, or other initial screening criteria, endoscopy has been placed on the right side of the algorithm apart from the main flow of diagnosis and treatment. By this positioning, the committee intends to indicate clearly that endoscopy should be used only as an adjunct modality in the diagnosis of FD.

The second feature is the changes made in the drugs used to treat FD. Depending on their recommendation grade and evidence level, such drugs are designated as either first- or second-line treatments. The first-line treatments are acid inhibitors, the prokinetic acotiamide, and the Japanese herbal medicine rikkunshito, and the second-line treatments are anxiolytics, antidepressants, prokinetics other than acotiamide, and herbal medicines other than rikkunshito. Acotiamide and rikkunshito have been classified as first-line treatments because the evidence for them is much stronger than the evidence for other prokinetics and herbal medicines, respectively.

The previous version of these clinical practice guidelines had separate algorithms for primary care physicians and gastrointestinal (GI) specialists [1], but at the beginning of the project to prepare the revised guidelines, the members of the Guidelines Creating Committee agreed to prepare guidelines that would be suitable for use by non-specialists. Therefore, in the revised guidelines, the diagnostic flow has been expressed in a single unified algorithm that we expect to be clear and broadly useful to both primary care physicians and specialists. In addition, to clarify the tests that should be performed by GI specialists when necessary, versus those to be performed routinely by primary care physicians, we have provided the table below indicating the diagnostic tests for FD to be used at different levels of clinical practice (Table 1).

**Table 1:**
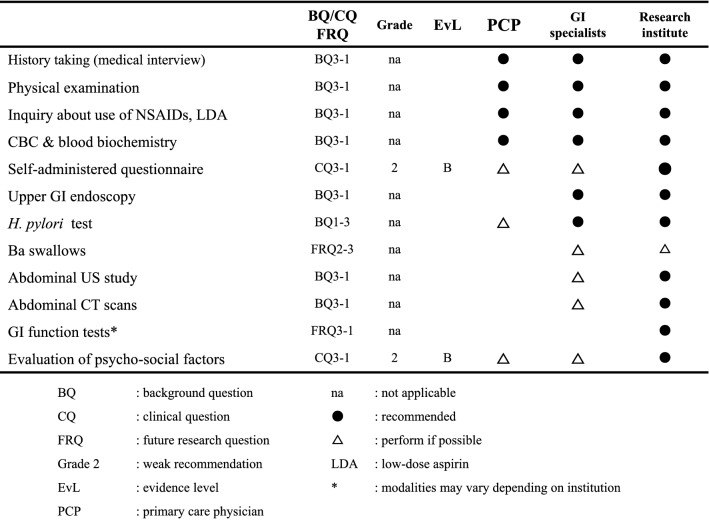
Diagnostic tests for FD depending on level of clinical practice

## Definition and epidemiology

### Functional dyspepsia, chronic gastritis, *Helicobacter pylori*-associated dyspepsia

- Functional dyspepsia (FD) is defined as a condition chronically presenting symptoms centered in the upper abdomen, such as epigastric pain or discomfort, in the absence of any organic, systemic, or metabolic disease that is likely to explain the symptoms.
- FD is defined by symptoms and chronic gastritis is defined by histological inflammation; therefore, these conditions are different. However, many FD patients have been treated as having chronic gastritis.
- Dyspepsia accompanied by *H*. *pylori* infection should be treated as *H*. *pylori*-associated dyspepsia.

*Comment:* in the Rome III criteria, FD is defined as the presence of one or more of four symptoms—postprandial fullness, early satiation, epigastric pain, and epigastric burning—that is unexplained after a routine clinical evaluation [2]. The Rome IV criteria, published in 2016, retain this definition [3]. In clinical settings, however, symptoms vary, with many patients complaining of symptoms other than the above-mentioned four. Therefore, these guidelines entrust the physicians who actually treat the patients to determine whether the patients’ complaints are dyspepsia symptoms. Likewise, the Asian consensus on FD does not restrict FD symptoms to the four specified in the Rome III and IV criteria [4]. As for symptom duration, the Rome IV criteria specify that FD symptoms must have been present for the 3 months before diagnosis and symptom onset must have preceded diagnosis by at least 6 months [3]. Most Japanese patients, however, do not meet those criteria for symptom duration because they generally visit a medical facility within a month of symptom onset, perhaps because almost all Japanese people have health insurance coverage. Therefore, in these guidelines, the meaning of “chronically” is not defined as a specific duration, but rather is left to the judgement of the physician treating the patient. By aligning the guidelines with clinical practice in this way, we expect that the disease name “functional dyspepsia” will be widely accepted and used by physicians in Japan.

Until recently, most FD patients in Japan have been diagnosed with and treated for chronic gastritis. However, chronic gastritis intrinsically involves histological inflammation of the gastric mucosa, and the diagnosis is unaffected by the presence or absence of symptoms. Gastritis is thus in a completely different diagnostic class from FD, which is diagnosed from symptoms. The use of these two quite different names for the diagnosis of the two conditions should help to reduce confusion.

Although the mechanism by which *H. pylori* infection affects gastro-duodenal pathophysiology remain unclear, eradication treatment for *H. pylori* improves dyspeptic symptoms in a subset of FD patients [5–7]. At the Kyoto Global Consensus Meeting for *H. pylori* Infection, which was held in 2014, the new entity “*H. pylori*-associated dyspepsia” was defined. It was decided that symptom improvement 6 months to 1 year after successful eradication identifies *H. pylori* as the organic cause of the symptoms and provides the rationale to consider *H. pylori*-associated dyspepsia as a separate clinical entity [8]. This concept is also supported by Rome IV [3]. However, the physician should not wait to start treatment for FD until several months after eradication. If patients complain of symptoms, treatment should be started immediately after eradication to improve the patients’ QOL.

### Prevalence of FD

- The prevalence of FD in Japanese patients ranges from 11 to 17% in patients who appear for medical checkups and from 45 to 53% in patients who seek medical care because of upper gastrointestinal symptoms.
- Because of the absence of reliable data, it is difficult to determine whether the prevalence of FD is increasing in Japan.

*Comment:* although the results have varied according to the definition of FD used in each study, the prevalence of FD in Japan has been found to be 11–17% in patients undergoing routine medical checkups and 45–53% in patients visiting a healthcare facility complaining of upper abdominal symptoms [1]. These prevalences are thought to be comparable to or lower than those in Western countries, but the Japanese data were all collected in single-center, cross-sectional studies, not multi-center epidemiological surveys. A web survey in the general population in Japan found that the prevalence of FD was 7% [9].

In a study from Japan evaluating the changes in endoscopic findings and symptoms over 25 years, the most common complaint throughout the period was “discomfort and/or pain”, while over time, the occurrence of normal endoscopic findings and the occurrence of erosive esophagitis increased [10]. These results suggest that the proportion of patients with upper abdominal symptoms accounted for by FD is increasing. Since the prevalence of FD varies depending on the definition and the patient population studied, it is difficult to evaluate accurate prevalence with the change of the times.

### Clinical characteristics of persons susceptible to FD

- Gene polymorphisms, childhood abuse, post-infectious gastroenteritis, female sex, and young age are related to FD, but no consensus has been obtained.
- Patient behavior with regard to clinic visitation is not influenced by the duration of FD but is influenced by symptom intensity.
- FD patients have impaired quality of life.

*Comment:* gene polymorphisms such as GNB3 825C>T, SCL6A4 5HTTLPR and CCK-1R 779T>C have been considered to be related to FD. A recent meta-analysis found that only the minor allele (T) in GNB3 825C>T was associated with an increased susceptibility to the epigastric pain syndrome subtype [11]. A population-based survey in Japan found that a history of physical, sexual, or psychological abuse in childhood was significantly prevalent in dyspepsia patients [12]. It is well known that FD develops after acute gastroenteritis, and a meta-analysis found that the odds ratio for development of post-infectious FD was 2.54 [13]. A 10-year population-based study found that FD was stable over the 10-year period and was more common in young subjects and females [14]. Since many of the studies referred to above were conducted in Western countries and the definitions of FD and study populations varied, one must exercise caution in applying the results to Japanese patients.

Examined in many studies, the clinic visitation behavior of FD patients has been found to be influenced by the frequency, duration, and severity of symptoms. Two studies from Japan suggested that patient behavior with regard to the first clinic visit was not influenced by the duration of FD [15, 16]. Another study from Japan indicated that anxiety, symptom intensity, the physical component summary of QOL, and overlap with epigastric pain syndrome (EPS) and postprandial distress syndrome (PDS) are significantly correlated with clinic visitation behavior [17].

Many studies have shown a clear correlation between severity of symptoms and negative impact on QOL. A study from Japan also found that FD patients showed significantly poorer health-related QOL across all domains compared with controls [18]. A large-scale, population-based study found that participants with FD had significantly greater health impairment and health-care usage than those without dyspepsia, and that participants with the overlapping variant showed greater somatization and poorer QOL scores than did individuals with either PDS or EPS alone [19]. A study from Japan found that patients with PDS, EPS, or EPS-PDS overlap had significantly lower QOL than the controls, but no difference was found among the subtypes [20]. There still have been only a small number of studies from Japan, so additional evidence is desired.

### FD and gastroparesis

- FD and gastroparesis are different disorders, but they are thought to overlap often.

*Comment:* gastroparesis (GP) is a disorder in which delayed gastric emptying occurs without any obstructive mechanism. GP is characterized by a combination of cardinal symptoms (nausea, vomiting, abdominal pain, early satiety, fullness, bloating) with no evidence of mechanical obstruction during gastroscopy, and a delayed 4-h solid-phase gastric-emptying scan [21]. Most cases of GP are idiopathic, but the disorder is also known to be associated with diabetes mellitus, gastric surgery, systemic disorders (e.g., chronic renal failure, Parkinson disease, scleroderma), drugs (e.g., opioids, anticholinergics) and viral infection [22, 23]. Pathologically, GP is simply delayed gastric emptying, whereas FD can involve delayed or accelerated gastric emptying, impaired gastric accommodation, and visceral hypersensitivity. Accordingly, GP, which is defined as delayed gastric emptying with or without comorbidity, is basically different from FD, but FD patients with delayed gastric emptying are thought to have overlapping idiopathic GP, the prevalence of which seems to be 10–20% of FD cases [24–26]. Because of the lack of a standardized diagnostic method, GP has not been adequately diagnosed in clinical practice.

## Pathophysiology

### Impaired gastric motility and visceral hypersensitivity

- Multiple factors contribute to the pathophysiology of FD.
- Disturbances of gastric accommodation, gastric emptying and gastroduodenal motility are involved in the pathogenesis of FD.
- Visceral hypersensitivity is involved in the pathogenesis of FD.

*Comment:* multiple factors may be associated with the pathophysiology of FD. These include impaired gastric accommodation, delayed gastric emptying [27, 28], visceral hypersensitivity, gastric acid, genetics, early-life events, lifestyle, microinflammation in the duodenum, and prior infection.

A close relationship between symptoms and impaired gastric accommodation in FD patients was found in a randomized, double-blind, placebo-controlled study [29]. Several reports have suggested that gastric emptying is impaired in some FD patients, and a meta-analysis indicated that it is significantly delayed in almost 35% of FD patients [26].

### Psychosocial factors and gastric acid

- The presence of gastric acid is thought to be a cause of FD.
- Psychosocial factors contribute to FD symptoms.

*Comment:* the efficacy of acid blockers for dyspeptic symptoms has been demonstrated in some meta-analyses. Additionally, acid infusion into the stomach induced dyspeptic symptoms in healthy Japanese control subjects, and those symptoms significantly increased in patients with FD [30]. Gastric and duodenal hypersensitivity to gastric acid are associated with FD symptoms [31]. Anxiety as a psychosocial factor evaluated by the Hospital Anxiety and Depression Scale is associated with uninvestigated FD.

### Genetics and early-life events

- It is possible that family history and genetic polymorphisms are associated with FD.
- A history of abuse in childhood and/or adolescence is associated with FD.

*Comment:* many studies have reported associations between risk of FD and genetic polymorphisms [32]. A history of abuse in childhood is associated with FD and the severity of FD symptoms in Japan [12].

### Post-infectious FD and microinflammation

- Post-infectious FD is also observed in Japan as well as other countries.
- Microinflammation of gastroduodenal mucosa is associated with FD.

*Comment:* there are data about post-infectious FD in Japan as well as other countries [13, 33]. Signs of microinflammation in the duodenum, such as the presence of eosinophils and mast cells, have been reported in patients with FD [34, 35]. Impaired duodenal mucosal integrity has also been associated with duodenal microinflammation in patients with FD [36].

### Lifestyle

- Lifestyle factors such as insufficient exercise, sleep disorders, high fat intake, and irregular eating patterns are involved in the pathophysiology of FD.

*Comment:* sleep disorders and insufficient exercise are associated with FD [37]. Fat intake aggravates clinical symptoms of FD, and irregular eating patterns are also associated with FD [38].

### Future research questions

### Pancreatic enzyme abnormalities and pancreatic dysfunction

- There are small but certain population of FD patients with pancreatic enzyme abnormalities or exocrine pancreatic dysfunction. It is still unknown whether pancreatic enzyme abnormalities and exocrine pancreatic dysfunction directly explain FD symptoms.

*Comment:* refractory FD patients should be further examined using endosonography in the view of the strategy for the treatment of early chronic pancreatitis [39, 40], because it is difficult to differentiate early chronic pancreatitis patients from FD patients with pancreatic enzyme abnormalities by clinical characteristics [41].

### Microbiota and food allergies

- It is possible that gastric and intestinal microbiota are involved in the pathophysiology of FD.
- There is little available data about food allergies in FD patients.

*Comment:* although intestinal microbiota have been reported to be associated with irritable bowl syndrome (IBS), there have been few reports about the relationship between FD and intestinal microbiota [42–44]. The relationships between food allergies and inflammatory cell infiltration in the gastroduodenal mucosa of FD patients are controversial [45, 46].

### Cascade stomach and gastroptosis

- Although there have been a few reports that cascade stomach and gastroptosis is associated with dyspepsia, their relationship with FD has not been clarified.

*Comment:* gastroptosis is less associated with dyspepsia, and cascade stomach is tend to be associated with FD symptoms [47].

## Diagnosis

### Upper gastrointestinal endoscopy

- Upper gastrointestinal endoscopy is not required to diagnose FD. FD should be diagnosed on the basis of a comprehensive evaluation of symptoms, age, medical history, presence of *H. pylori* infection, and laboratory history. However, endoscopy or other investigations should be performed when organic disease is suspected because of a positive alarm sign.

*Comment:* if there are no alarm signs and no suspicion of other organic disease, endoscopy is not necessary, and FD treatment should be started [4, 48–50]. New onset of symptoms at an advanced age, weight loss, recurrent vomiting, bleeding, dysphagia, painful swallowing, abdominal mass, fever, and family history of esophageal or gastric cancer should be considered alarm signs for the presence of organic disease. Since currently there are no effective biomarkers for the diagnosis of FD, if an alarm sign is noted, a thorough examination including blood sampling, upper gastrointestinal endoscopy, and other diagnostic imaging (abdominal ultrasonography, abdominal computed tomography, etc.) should be performed to check for organic disease. Diseases and histories that can cause symptoms associated with FD include malignant diseases such as gastric cancer, esophageal cancer, and pancreatic cancer; inflammatory diseases such as reflux esophagitis, gastric and duodenal ulcer, chronic pancreatitis, and chronic cholecystitis; metabolic endocrine diseases such as diabetes mellitus and thyroid diseases; drug-induced diseases caused by nonsteroidal anti-inflammatory drugs and low-dose aspirin; and a history of abdominal surgery. If there are signs suggestive of such diseases, endoscopy and any other tests necessary to exclude them should be performed, as in the case of alarm signs. However, the absence of alarm signs does not exclude the possibility of organic disease. If the patient does not respond to the initial treatment, or the symptoms flare up after discontinuation of the treatment, it is important to conduct a thorough examination for organic disease.

### Self-reporting questionnaires

- A self-reporting questionnaire is useful for the diagnosis of FD. [Recommendation Weak (92%), evidence level B].

*Comment:* self-reporting questionnaires are used to objectively evaluate the type and degree of FD symptoms. Self-administered questionnaires include the Gastrointestinal Symptom Rating Scale (GSRS) [51], the Global Overall Symptom (GOS) scale [52], the Izumo scale [53], the Frequency Scale for the Symptoms of Gastroesophageal Reflux Disease (FSSG; F scale) [54], and pictograms that use illustrations to more clearly indicate the quality and location of symptoms [55], all of which have been reported to be effective. Such self-administered questionnaires are very useful not only for initial diagnosis but also for follow-up observation and judging the effectiveness of FD treatment.

Psychosocial factors are thought to be involved in the pathogenesis and pathophysiology of FD. The self-administered questionnaires are insufficient for understanding these factors, but the Rome IV Psychosocial Alarm Questionnaire for functional gastrointestinal disorders (FGIDs), published by the Rome Committee on Psychosocial Factors, can be used for psychological screening.

Although self-administered questionnaires alone cannot be used to diagnose FD, their use is recommended because they are considered to be highly useful in both the diagnosis and treatment of FD.

### Gastrointestinal function testing

- The usefulness of gastrointestinal function tests in clinical practice is not clear. Such tests are not widely available, and their results do not necessarily agree with pathogenesis or improve therapeutic predictability for functional gastrointestinal disorders. However, they may become a powerful diagnostic tool for classifying FD into clinically meaningful subtypes.

*Comment:* since the pathogenesis of FD includes visceral hypersensitivity and abnormalities in gastric and duodenal motility, evaluation of those phenomena is useful for clarification of FD pathogenesis. Gastrointestinal motility can be evaluated by pressure measurements in the gastrointestinal tract, electrogastrography, barostat testing, radioisotopic testing of gastric evacuation, expiration testing of gastric evacuation capacity, and ultrasonography of gastric evacuation and duodenogastric reflux, while visceral hypersensitivity can be evaluated by barostat testing and the water-drinking test [56]. A meta-analysis found that delayed gastric emptying in gastric emptying tests is associated with upper gastrointestinal symptoms [57]. Some study results have shown delayed gastric emptying in normal subjects and FD patients, while others have shown no significant difference, so the clinical usefulness of this test in diagnosis of FD has not been established [58, 59]. Because the presence or absence of gastrointestinal dysfunction does not necessarily correlate with pathology or response to treatment, the American College of Gastroenterology guidelines and the Asian consensus for FD state that gastrointestinal function testing is not recommended as a routine clinical procedure. Such testing can clarify the pathogenesis of symptoms such as delayed gastric emptying and gastric fundic accommodation disorder in some cases, but can only be performed in limited facilities as part of clinical research.

## Treatment

### Background knowledge

- Satisfactory relief of symptoms is an important objective in the treatment of FD.
- Placebo may have a profound effect on FD symptoms.
- Establishing a good patient-physician relationship is useful in the treatment of FD.

*Comment*: “Satisfactory or adequate relief of symptoms” has been used as an acceptable primary endpoint in clinical trials to treat patients with FGIDs [60], and has also served as an appropriate endpoint in a clinical trial to treat patients with FD [61].

Placebo was highly effective as a treatment for FD, with the effect strongly influenced by the brain-gut interaction. A meta-analysis showed that the placebo effect ranges from 5 to 90%, with an average of 56% [62]. Factor analysis of the effect of the placebo treatment on FD symptoms showed that low body mass index, homeostatic symptoms, and smoking reduced the effect of the placebo treatment [63, 64].

Since psychological distress is an important risk factor for the development of FGIDs, the need to maintain a good patient-physician relationship and listen to the patient's psychosocial background from the initial consultation stage was described in the Rome IV criteria [65]. A good patient-physician relationship also improves patient satisfaction, treatment compliance, and treatment effectiveness.

## Clinical statement

### First-line treatment

- Lifestyle and dietary modifications are effective for FD. [Recommendation Strong (100%), evidence level B].

*Comment*: there were no prospective studies evaluating the efficacy of lifestyle and dietary modifications. However, Pilichiewicz et al. reported that a high-fat meal induced greater nausea and pain in FD patients than did a high-carbohydrate or control meal [66], which suggested that avoiding dietary fat might be beneficial for the treatment of FD. Furthermore, smoking has been associated with the presence of FD (odds ratio, 1.50) [67], which indicates that smoking cessation might also be an effective lifestyle modification.

- Proton pump inhibitors (PPIs) and histamine type 2 receptor antagonists (H2RAs) are effective for the treatment of FD. [Recommendation Strong (100%), evidence level A]
- The efficacy of potassium-competitive acid blockers (P-CABs) cannot be evaluated because of little evidence. [Recommendation Weak (77%), evidence level C]

*Comment*: in a clinical trial in Japan (the SAMURAI study), complete symptom relief was not different between the placebo and rabeprazole groups, but the satisfactory symptom relief of rabeprazole 20 mg was significantly higher than that of placebo (Table 2) [68]. The Cochrane Database Systematic Review in 2017 found that PPIs are effective in patients with FD (response rate, 31.1% vs. 25.8% for placebo), with the number needed to treat for an additional beneficial outcome being 11 (Table 3) [69]. These data may indicate the limit of effectiveness of acid suppressants for the treatment of FD. The Cochrane Database Systematic Review in 2006 found that H2RAs are effective in patients with FD (relative risk reduction, 23% vs. placebo) [62]. The efficacy of P-CABs over placebo has not been proven in a randomized, controlled trial.

- The acetylcholinesterase (AChE) inhibitor acotiamide is useful, and its use is recommended. [Recommendation Strong (100%), evidence level A]

**Table 2:** Randomized double-blind clinical trials for FD in Japan

Author	Drug	Dose	Case number (*N*)	Duration (weeks)	Primary outcome	Primary endpoint	NNT (*N*)
Miwa 2006 [80]	Rebamipide	300 mg	81	4	Dyspeptic symptom	Negative	100
Miwa 2009 [78]	Tandospirone	30 mg	150	4	Abdominal symptom	Positive	6
Matsueda 2010 [70]	Z-338 (Acotiamide)	300 mg	462	4	Overall treatment efficacy	Negative	11
Matsueda 2012 [95]	Acotiamide	300 mg	1394	4	Overall treatment efficacy and elimination rate of symptom	Positive	6
Suzuki 2013 [96]	Lansoprazole	15 mg	54	4	Overall dyspeptic symptom relief rate	Positive	5
Iwakiri 2013 [68]	Rabeprazole	20 mg	392	8	Complete relief of symptom	Negative	9
Suzuki 2014 [97]	Rikkunshito	7.5 g	247	8	Responder rate	Negative	11
Ohtsu 2017 [98]	*Lactobacillus gasseri OLL2716*	85 g	116	12	Overall treatment efficacy	Negative	7
Tominaga 2018 [73]	Rikkunshito	7.5 g	192	8	Overall treatment efficacy	Positive	7

*NNT* number needed to treat

**Table 3:** Representative systematic reviews and meta-analyses of treatments for FD

Drug	Number of studies (*N*)	Number of participants (*N*)	NNT (N)
*H. pylori* eradication^a^ [7]	18	3970	13
Proton Pump Inhibitors [69]	18	6172	11
Psychotropic drugs [77]	13	1241	6
Prokinetics [71]	29	10,044	7
Acupuncture [83]	7	542	N/A

^a^Patients who remain symptom-free after eradication are now considered as *H. pylor*i-associated dyspepsia

*NNT* number needed to treat

*Comment*: a clinical trial in Japanese subjects (Table 2) [70] and a systematic review found that acotiamide was more effective than the control in the treatment of overall symptoms of FD and PDS (Table 3) [71].

- The Japanese herbal medicine rikkunshito is an effective treatment for FD, and its use is recommended. [Recommendation Strong (92%), evidence level A].

*Comment*: there is various evidence that rikkunshito, a Japanese herbal medicine, improves functional disorders of the gastrointestinal tract [72]. A recent randomized clinical trial (the DREAM study) found that the therapeutic efficacy of rikkunshito for dyspepsia correlated with improvement in anxiety in patients with FD (Table 2) [73].

### Second-line treatment

- Dopamine receptor antagonists are useful, and their use is suggested. [Recommendation Weak (85%), evidence level B]
- Serotonin-4 (5-HT4) receptor agonists are useful, and their use is suggested. [Recommendation Weak (85%), evidence level B]

*Comment:* there are no placebo-controlled studies for metoclopramide or domperidone. Compared with the control, itopride was associated with lower overall patient ratings, lower postprandial bloating, and lower early satiety [74]. In a randomized, open-label study in Japanese patients (Japan Mosapride Mega-Study), mosapride was significantly effective in improving symptoms [75].

- Herbal medicines other than rikkunshito may be effective for the treatment of FD, and their use is suggested. [Recommendation Weak (100%), evidence level B].

*Comment:* herbal medicines other than rikkunshito may prove to be useful in treating FD, but the evidence available at present is insufficient for their use to be strongly recommended.

- Tricyclic antidepressants and anxiolytics such as tandospirone are effective for the treatment of FD and have been proposed for use in the treatment of FD patients. [Recommendation Weak (92%), evidence level A for tricyclic antidepressants and B for anxiolytics such as tandospirone]

*Comment*: two meta-analyses have shown the efficacy of tricyclic antidepressants (TCAs) in the treatment of FD patients (Table 3) [76, 77], but TCAs should be used carefully because of their side effects. The meta-analysis conducted by Ford et al. did not find serotonin-1A (5-HT1A) receptor agonists to be effective in the treatment of FD [77], but a Japanese randomized, controlled trial with sample size of 150 subjects found that the 5-HT1A agonist tandospirone citrate was effective in the treatment of FD (Table 2) [78].

### Alternative or complementary therapy

- It is not clear whether antacids, prostaglandin analogs (e.g., misoprostol), or gastroprotective agents (e.g., sucralfate and rebamipide) are effective treatments for FD. [Recommendation NA, evidence level B].
- The implementation of psychosomatic internal medical treatment has been proposed because it effectively treated FD. [Recommendation Weak (100%), evidence level B].

*Comment*: The Cochrane Database Systematic Review in 2006 found no effect for the above-mentioned drugs [62]. Although the efficacy of rebamipide was assessed in double-blind, placebo-controlled studies, one study from the United States was terminated before it reached the planned sample size [79], and one study from Japan showed no effect (Table 2) [80], suggesting that the effectiveness of this drug is not yet clear.

In a study of cognitive-behavioral therapy (CBT) as a treatment for FD, Haug et al. reported that compared with the control group, the CBT group had a shorter duration of epigastric pain and showed alleviation of nausea and heartburn [81]. Furthermore, in a study that compared drug treatment plus CBT with drug treatment alone in patients with FD, Orive et al. found that at the end of the 10-week treatment period, the severity of symptoms improved more in the CBT-plus-drug group than in the drug-alone group [82].

### Future research themes

- The number of reports of combination therapy is increasing, but further evidence is needed.
- Some studies have found acupuncture to be an effective treatment for FD.
- The efficacy of moxibustion as a treatment for FD is unknown because of little evidence.
- Subtype-based treatment of FD is controversial.
- It is recommended that treatment of refractory FD be changed after 4–8 weeks, but further investigation is needed.

*Comment*: in the Rome IV criteria, there is no mention of combination drug therapy for FD [3]. However, there are five reports on the efficacy of combination drug therapy, which may be an alternative option for symptom improvement in patients with gastroesophageal reflux disease complicated by FD.

The Cochrane Database Systematic Review in 2014 found that acupuncture had some efficacy as a treatment for FD, but the evidence level of the clinical studies in the review was mostly low (Table 3) [83]. However, in 2020, a randomized, controlled trial in over 200 patients with FD found that acupuncture showed therapeutic efficacy compared with sham acupuncture [84]. Although acupuncture and moxibustion are both widely practiced in Japan, no clinical studies with a high evidence level of either of them as a treatment for FD have been reported from Japan.

Subtype-based treatment of FD was proposed in the Rome IV criteria, with the recommended first-line treatment being acid suppressants such as PPIs [3] for patients with EPS and prokinetics for patients with PDS. Since then, however, use of subtype-based treatment options has not been supported by sufficient evidence, so the joint American and Canadian guidelines on dyspepsia management published in 2017 did not recommend using subtype classification to guide treatment choice [48].

Because there is no definition of refractory FD, the appropriate time to change FD treatment in patients with refractory FD is not known. The first edition of the Japanese clinical practice guidelines for FD recommended that the treatment regimen of patients with refractory FD be changed after 4 weeks of treatment [1]. The above-mentioned joint American and Canadian guidelines recommend secondary treatment for refractory FD after 8 weeks of first-line treatment with PPIs [48]. A supportive meta-analysis of factors affecting the placebo response rate in IBS found a gradual decrease in the placebo effect when the treatment duration was longer than 4 weeks [85].

### Prognosis and complications

- FD sometimes recurs, but recurrence is not associated with an increased mortality.
- Irritable bowel syndrome, gastroesophageal reflux disease, chronic constipation, and other disorders often overlap with FD.

*Comment:* there are reports indicating that FD can recur. In a European study of patients with FD, dyspepsia symptoms recurred within 3 months in 20% of the patients whose symptoms had disappeared after 4 weeks of treatment with a PPI or placebo [86]. Similarly, in a Japanese study of patients with FD whose symptoms improved with acotiamide therapy, FD was found to have recurred in 25% of the patients at 1 year [87].

In a population-based cohort study in the United States of the impact of FGIDs on survival with over 30,000 person-years of follow-up, no association with overall survival was detected for dyspepsia, IBS, chronic diarrhea, or abdominal pain; only chronic constipation was related to poorer survival [88]. Likewise, a population-based cohort study in the United Kingdom with over 84,000 person-years of follow-up also found that dyspepsia did not increase mortality [89]. Taken together, these studies indicate that FD does not appear to increase mortality.

FGIDs often overlap with FD, with IBS being the FGID that does so most frequently (66.9% of FD cases, as diagnosed by the Rome IV criteria). Furthermore, FD with PDS is likely to overlap IBS with constipation.

Reflux esophagitis and non-erosive reflux disease frequently overlap FD [15]. A study using pH monitoring found that 23% of FD patients showed abnormal reflux [90]. Furthermore, functional heartburn overlaps with FD more frequently than does non-erosive reflux disease [91]. In a meta-analysis, individuals with weekly gastroesophageal reflux symptoms showed a high odds ratio (6.94) for dyspepsia [92]. Overlap of functional constipation (FC) was 39.0% in FD diagnosed by the Rome IV criteria [93], and in a Japanese study, overlap of FC occurred in 13.8% of FD patients diagnosed by the Rome III criteria [94]. Chronic pancreatitis may not be completely excluded from FD. Reports indicate that anxiety and depression overlap with FD. When FD patients have overlapping symptoms of other FGIDs and anxiety, their health-related quality of life worsens.

Although other diseases, including FGIDs, often overlap FD, the prevalence is easily affected by the diagnostic criteria and the population assessed, so those factors should be taken into consideration when interpreting data on overlapping FD.

## Appendix

The members of the Guidelines Committees who created and evaluated the Japanese Society of Gastroenterology “Evidence-based clinical practice guidelines for functional dyspepsia” are listed below.

### Creation Committee

Chair: Hiroto Miwa (Division of Gastroenterology and Hepatology, Department of Internal Medicine, Hyogo College of Medicine). Vice-Chair: Akihito Nagahara (Department of Gastroenterology, Juntendo University School of Medicine). Members: Akihiro Asakawa (Division of Psychosomatic Internal Medicine, Department of Social and Behavioral Medicine, Kagoshima University Graduate School of Medical and Dental Sciences), Makoto Arai (Department of Gastroenterology, Tokyo Women’s Medical University Yachiyo Medical Center), Tadayuki Oshima (Division of Gastroenterology and Hepatology, Department of Internal Medicine, Hyogo College of Medicine), Kunio Kasugai (Division of Gastroenterology, Department of Internal Medicine, Aichi Medical University), Kazuhiro Kamada (Department of Gastroenterology and Hepatology, Kyoto Prefectural University of Medicine), Hidekazu Suzuki (Division of Gastroenterology and Hepatology, Department of Internal Medicine, Tokai University School of Medicine), Fumio Tanaka (Department of Gastroenterology, Osaka City University Graduate School of Medicine), Kazunari Tominaga (Department of Gastroenterology, Hoshigaoka Medical Center), Seiji Futagami (Division of Gastroenterology, Department of Internal Medicine, Nippon Medical School), Mariko Hojo (Department of Gastroenterology, Juntendo University School of Medicine), Hiroshi Mihara (Third Department of Internal medicine, University of Toyama).

### Evaluation Committee

Chair: Kazuhide Higuchi (The Second Department of Internal Medicine, Osaka Medical and Pharmaceutical University). Vice-Chair: Motoyasu Kusano (Department of Gastroenterology and Hepatology, Gunma University Graduate School of Medicine). Members: Tomiyasu Arisawa (Department of Gastroenterology, Nagoya Kyoritsu Hospital), Mototsugu Kato (Department of Gastroenterology, National Hospital Organization Hakodate National Hospital), Takashi Joh (Gamagori City Hospital).

### The Japanese Society of Gastroenterology

President: Kazuhiko Koike (Kanto Central Hospital). Past President: Tooru Shimosegawa (South Miyagi Medical Center). Directors Responsible: Satoshi Mochida (Department of Gastroenterology & Hepatology, Saitama Medical University), Nobuyuki Enomoto (First Department of Internal Medicine, Faculty of Medicine, University of Yamanashi).
